# Dorsal and medial raphe nuclei participate differentially in reproductive functions of the male rat

**DOI:** 10.1186/s12958-015-0130-0

**Published:** 2015-12-08

**Authors:** María E. Ayala, Diana E. Velázquez, Juan L. Mendoza, Juana Monroy, Roberto Domínguez, Mario Cárdenas, Andrés Aragón

**Affiliations:** Unidad de Investigación en Biología de la Reproducción, Laboratorio de Pubertad, Facultad de Estudios Superiores Zaragoza, UNAM. AP 9-020, CP 15000, Distrito Federal, México; Laboratorio de Hormonas Proteicas, Instituto de Ciencias Médicas y de la Salud Salvador Subirán, Tlalpan, CP 14000 México, D.F. México; Ciencia, Tecnología e Informática Aplicadas a la Reproducción S A, Texcoco, CP 56200, Estado de México, México; Instituto Tecnológico del Altiplano de Tlaxcala, San Diego Xocoyucan, CP 90122, Tlaxcala, México

**Keywords:** Serotonin, hypothalamus, gonadotropin, seminiferous epithelium, sperm

## Abstract

**Background:**

Innervation of the hypothalamus and median eminence arise from the dorsal and medial raphe nuclei (DRN and MRN, respectively). The hypothalamus regulates the secretion of gonadotropins, which in turn regulate the reproductive function of males and females. However, it is not known the role of raphe nuclei in male reproductive function. Our goal was to investigate the role of the DRN and MRN in the regulation of the testicular function and secretion of gonadotropins in prepubertal rats.

**Methods:**

Dihydroxytryptamine (5,6-DHT) in ascorbic acid was used to chemically lesion the DRN or MRN. Rats were treated at 30 days-of-age and sacrificed at 45 or 65 days-of-age. Sham-treated controls were injected with ascorbic acid only. Negative controls were untreated rats. The damage induced by the 5,6-DHT was monitored in coronal serial sections of DRN and MRN; only the animals in which lesion of the DRN or MRN was detected were included in this study. As output parameters, we measured the concentrations of noradrenaline (NA), dopamine (DA) and serotonin (5-HT) in the anterior (AH) and medial (MH) hypothalamus by high performance liquid chromatography (HPLC); whereas, circulating concentrations of gonadotropins and sexual steroids were measured by radioimmunoassay. Seminiferous epithelium and sperm quality were also evaluated.

**Results:**

Lesion of DRN or MRN does not induced changes in concentrations of LH, progesterone, and testosterone. Compared with the control group, the sham or lesion of the DRN or MRN did not modify noradrenaline or dopamine concentrations in the AH and MH at 45 or 65 days of age. Meanwhile, serotonin concentrations decreased significantly in lesioned rats. Lesion of DRN induced significantly lower concentrations of FSH regardless of age; similar lesion in the MRN had no impact on FSH levels. Sperm concentration and motility were significantly decreased in the same animals. The lesion of the MRN does not induced changes in the seminiferous epithelium or gonadotropin levels. Our results suggest that raphe nuclei regulate differentially the male reproductive functions.

**Conclusions:**

The DRN but not the MRN regulates the secretion of gonadotropins and testicular function.

**Electronic supplementary material:**

The online version of this article (doi:10.1186/s12958-015-0130-0) contains supplementary material, which is available to authorized users.

## Background

The regulation of testicular function depends on the integration of neuroendocrine mechanisms that involve the hypothalamus-hypophysis axis [1]. Gonadotropins FSH and LH, secreted by the hypophysis, regulate spermatogenesis. Gonadotropin secretion is modulated by gonadotropin releasing hormone (GnRH), which is produced by the hypothalamus [2]. Furthermore, the neural information from extrahypothalamic areas, such as the DRN and MRN raphe nuclei, regulates the secretion of gonadotropins in adult female [3–6] and male [7–9] rats.

Ascending serotonergic projections from the DRN and MRN innervate the hypothalamus and median eminence [8, 10–12] and synapses with GnRH neurons, suggesting the participation of serotonin on GnRH and gonadotropin secretion [13–15]. In adult male rats, LH secretion is unchanged after sectioning of fibers arising from the MRN. In contrast, LH secretion decreases after sectioning of fibers arising from the DRN [7], or increases following chemical lesion by injection of 5,7-dihydroxytryptamine (5,7-DHT) into the DRN [9]. These results cannot be attributed solely to the serotonergic system, because the DRN and MRN contain both serotonergic and non-serotonergic neurons [16–18]. Furthermore, 5,7-DHT is a non-specific neurotoxin, as it damages both serotonergic and noradrenergic neurons [19].

In the female, the serotonergic system, arising from the DRN and MRN, differentially participates in the regulation of gonadotropin secretion. This can be attributed to the complexity of these nuclei, because serotonergic neurons from the DRN and MRN exhibit distinct anatomical, electrophysiological, and functional properties [20–23]. These distinct features suggest different functional roles for the two serotonergic systems. Despite increased interest in the functional properties of these individual raphe nuclei, very little is known about the participation of the serotonergic system, originating from the raphe nuclei, in the regulation of testicular function in the adult male rat. Furthermore, no information exists on the participation of these nuclei during puberty in the male rat.

Some derivates of amphetamine exert differential neurotoxic effects on the serotonergic neurons from the DRN and MRN; the serotonergic neurons from the DRN are more affected than MRN cells [20, 21, 24]. The neurotoxic effects of these drugs on serotonergic neurons are related to the formation of toxic metabolites, such as 5,6-dihydroxytryptamine (5,6-DHT) [21, 25–27].

The objective of this work was to investigate the role of the serotonergic system from the DRN and MRN on the secretion of gonadotropins and reproductive function of male rats. We achieved our objective by lesioning, with 5,6-DHT, the serotonergic neurons of the raphe nuclei and evaluating the circulating gonadotropins and sexual steroids, seminiferous epithelium, and sperm quality.

## Materials and Methods

### Experimental strategy

Male rats of the CII-ZV strain (n = 160), from our own breeding stock, were maintained under controlled light conditions (lights on from 05:00 to 19:00), with free access to food and tap water. We allocated animals randomly to one of three groups described below. At 30 days of age, the MRN or DRN of prepuberal male rats was chemically lesioned. All animals were sacrificed at 45 or 65 days of age, 15 or 35 days after lesion, circulating concentrations of sexual steroids and gonadotropins, seminiferous epithelium, and sperm quality were evaluated.

Rationales for use of prepuberal animals and schedule of sampling were the permissive role of serotonin toward pubertal maturation, the age at which spermatogenesis is completed in the testis, and the time required for the sperm traverse the epididymis.

### Ethical approval

We conducted all experiments in strict accordance with the Mexican Law of Animal Treatment and Protection Guidelines. The Institutional Ethics Committee of the Facultad de Estudios Superiores Zaragoza, Universidad Nacional Autónoma de México approved the protocol (Letter date 01/03/2012).

### Control Group

The animals were untreated.

### Lesioned groups

Animals were anesthetized with sodium pentobarbital (Anestesal, Smith Kline Norden of Mexico, Monterrey Nuevo León; 35 mg kg^−1^ of body weight, i.p.) and placed in a stereotaxic apparatus. The head skin was opened, the skull drilled and a number 29-needle (13 mm long) introduced following parameters of the stereotaxic atlas by Paxinos and Watson [28]. Coordinates for the DNR are: AP = 0.7 mm from lambda, H = 3.3 mm, and L = 6.4 mm at a 30° angle in relation to its perpendicular axis from the dorsal raphe. For MRN: AP = 0.5 mm, H = 4.9 mm, and L = 8.0 mm at a 35° angle in relation to its perpendicular axis. To produce the lesion of the DRN or the MRN, the needle was connected via tubing to a 100 μl Hamilton syringe mounted on a microinjection pump (MCA/100 Bionalytical Systems, Inc., West Lafayette, In, USA). 10 μg of 5,6-DHT (Sigma Chemical Co., St Louis, Mo, USA) dissolved in 2.5 μl of 0.05 % ascorbic acid (pH 3.5) was injected at an infusion rate of 1 μl/min. 5,6-DHT is a serotonergic-specific neurotoxin [6]. The 5,6-DHT dose was selected based on previous experiments, because 10 μg generated a selective depletion of serotonin concentration in the hypothalamus (50 %) but does not impact catecholamine concentration [29].

### Sham-operated groups

Animals were treated with the same parameters used in the lesion groups, but were injected with 2.5 μl of 0.05 % of ascorbic acid; lesion of the DRN or the MRN.

### Autopsy Procedure

The animals were killed by bleeding under ether anesthesia. The brain was quickly removed and placed in cold saline solution and frozen in liquid nitrogen. After careful removal of the nerves and optic chiasm, the anterior and medium hypothalamus were dissected following the parameters described previously [28]. The anterior hypothalamus (Bregma 20.8 to Bregma 21.8) included the removal of the lateral and median pre-optic nuclei, the suprachiasmatic, the paraventricular, the periventricular, and the stria terminal pre-optic area. The medial hypothalamus (Bregma 22.3 to Bregma 23.2) included the median eminence and arcuate nucleus. Both hypothalamic regions were stored at −70 °C, until the concentrations of serotonin, and its metabolite 5-HIAA, were measured using HPLC.

Blood was collected from the trunk of the animals, allowed to clot, and subsequently centrifuged at 3000 rpm. The serum supernatant was collected and stored at −20 °C. We measured progesterone, testosterone, LH, and FSH levels as described below. The testes, epididymis, prostate, and seminal vesicles were dissected and individually weighed on a precision balance.

### Measurement of serotonin, noradrenaline, and dopamine

The amounts of serotonin (5-HT), noradrenaline (NA), and dopamine (DA), were measured using the methodology described by Monroy *et al*. [6]. Briefly, tissue samples were weighed and homogenized in 300 μl of 0.1 N perchloric acid and subsequently centrifuged at 12500 rpm for 30 min at −4 °C. The supernatant was filtered using 0.2 μm regenerated cellulose filters, and 20 μl of this extract was injected into the high-performance liquid chromatography system for analysis. The results are expressed as ng/mg of weight of tissue.

### Hormone determination

Serum concentrations of progesterone and testosterone were measured by specific radioimmunoassay (RIA), using kits purchased from Diagnostic Products (Los Angeles, CA, USA). The intra- and inter-assay coefficients of variation were 5.3 and 9.87 % for progesterone and 4.3 and 7.8 % for testosterone. The detection limits for progesterone and testosterone were 0.09 and 0.005 ng/ml, respectively. The results are expressed as ng/ml.

Concentrations of LH and FSH were measured by RIA, with the reagents and protocol supplied by the National Hormone and Pituitary Programs (Baltimore, MD, USA). The values are expressed in ng/ml in terms of the reference preparations of NIH LH-RP2 and FSH-RP2. The intra- and inter-assay coefficients of variation were 7.91 % and 5.74 % for LH and 9.3 and 6.82 % for FSH. The detection limits for LH and FSH were 0.05 and 0.01 ng/ml, respectively.

### Sperm quality

Evaluation of sperm quality was performed in animals of 65 days-of-age, because at this age male rats have mature sperm present in the vas deferens. The content of vas deferens was obtained and placed in 2 ml of Tyrode’s salt solution (Sigma Chemical Co., St Louis, Mo, USA) at 37 °C and gently mixed to homogenize the suspension. A drop of the sperm suspension was placed on a slide for evaluation of motility. For each animal, 200 spermatozoa were analyzed [30, 31] at 100x total magnification using a phase-contrast microscope (Nikon, Tokyo, Japan). The percentage of motile sperm was defined as the ratio of cells showing movement to the total number of cells x 100. Sperm viability was determined using 1 % trypan blue (Sigma Chemical Co., St Louis, Mo, USA) following the technique described [30] and 200 cells were analyzed for each animal; results are expressed as percentage of viability. Sperm concentration was determined in a Neubauer chamber (PROPER Lumycite, 1/100 mm depth), results are presented as 1x10^6^ cells/ml.

### Analysis of seminiferous epithelium

The right testis of each animal was fixed by immersion in Bouin solution, followed by dehydration and embedding in paraffin wax. Serial 10 μm sections were obtained and stained with periodic acid Schiff (PAS)-hematoxylin.

From each animal, fifty seminiferous tubules, approximately round, were taken randomly and two diameters were measured: the maximum diameter, and the diameter perpendicular to the maximum. The following formula was used to estimate the area: area = πr^2^, where r = (diameter one + diameter two)/4. Results are presented as mean ± sd of area in each experimental group.

Staging was realized for each tubule evaluated for area; it was realized by following the development of the acrosome, as reported previously [32]. Each tubule staged was searched for gross histopathological changes. Results are presented as mean ± se, of percentage from each stage of seminiferous epithelium, in each experimental group.

### Brain histology

**Fig. 1:**
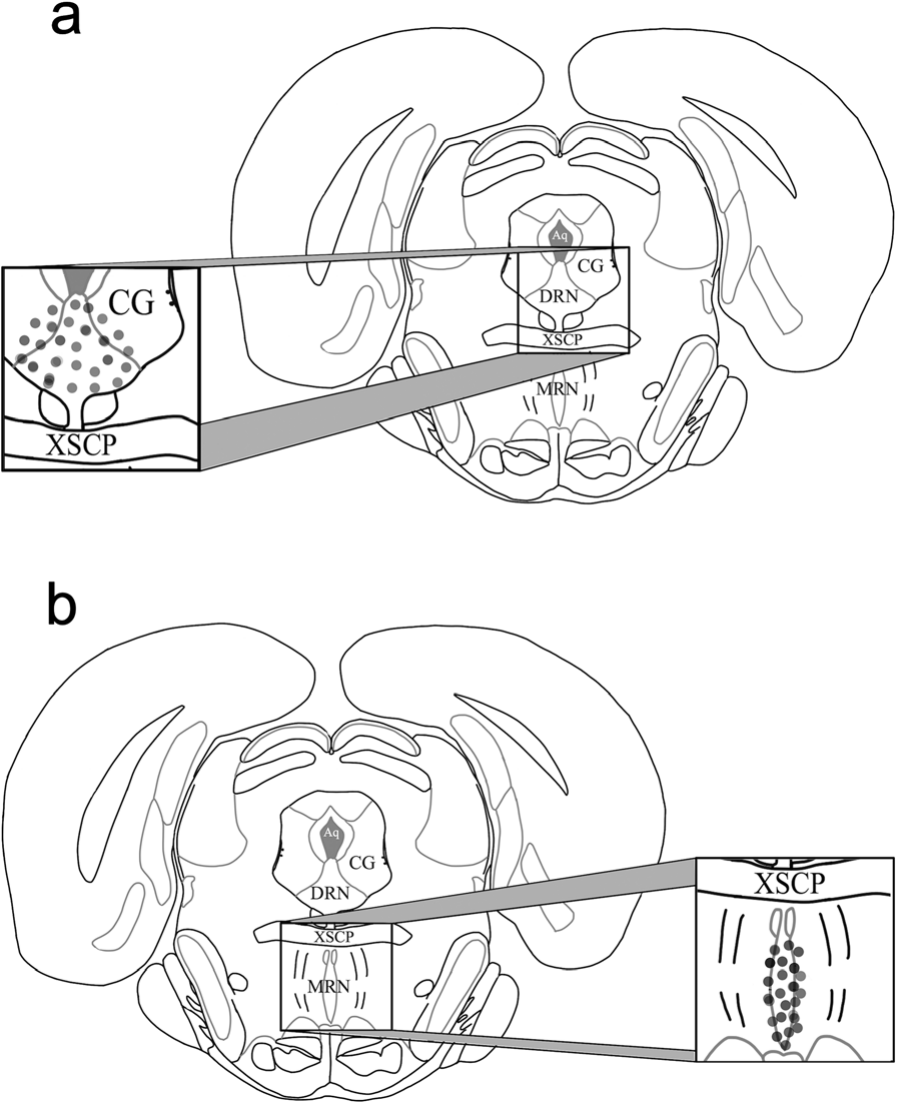
Diagrammatic representation of location of lesion sites. Dorsal (**a**) or medial raphe nuclei (**b**) on coronal sections (Bregma −7.8 mm). The area filled with gray circles represents the extent of the lesion. The figure was created on the basis of the Atlas of Paxinos and Watson [28]. Abbreviations: Aq, cerebral aqueduct; CG, central gray; XSCP, decussation superior cerebellar peduncle

After dissecting the hypothalamus, the caudal portion of the brain was fixed in 10 % formalin for at least 48 h. Brains were frozen and sectioned serially at 40 μm in the coronal plane using a cryostat kept at −20 °C. The sections were placed on glass slides and later stained with cresyl violet (Sigma Chemical Co., St Louis, Mo, USA) to confirm the trajectory of the needle, location of the injection site (Fig. 1a, b), and extent of the lesion. Based on the histological examination of the lesions, only animals with complete lesion of the DRN or MRN were included in this study.

The lesion of the DRN or MRN was determined by light microscopy. The damaged area was unstained, while the healthy tissue was stained violet.

The lesion of the DRN or MRN (Fig. 1) was considered complete when the injured area included the rostral and medial region of the nucleus, and the caudal region was not affected. Also, the dorsal, ventral, and lateral subdivisions of the nucleus were damaged with less destruction of the adjacent area, the *substantia grisea periventricularis*.

### Statistical analysis

The normality of the data was corroborated graphically in different experiments by checking residuals. The body and organ weights, sperm motility, sperm viability, serum concentration of hormones, as well as serotonin, noradrenaline and dopamine concentrations were analyzed by two-way ANOVA, considering chemical treatment as factor and age of animals as block. Tukey test was used as *post hoc* test. Proportions of stages of seminiferous epithelium, among treatments, were tested for independence by Pearson Chi-squared test. Comparison of the area of seminiferous tubules, in each stage of cycle of seminiferous epithelium, was realized by one way ANOVA for unbalanced models, followed by linear contrast of means. The results were considered significant at the *P* < 0.05 level. All analysis were realized with R version 3.1.2 [33] and packages car [34], vcd [35–37], rattle [38], and lattice [39], running on a MacBook with MacOsX version 10.10.1.

## Results

### Effects of the sham operation on the DRN or MNR

**Fig. 2:**
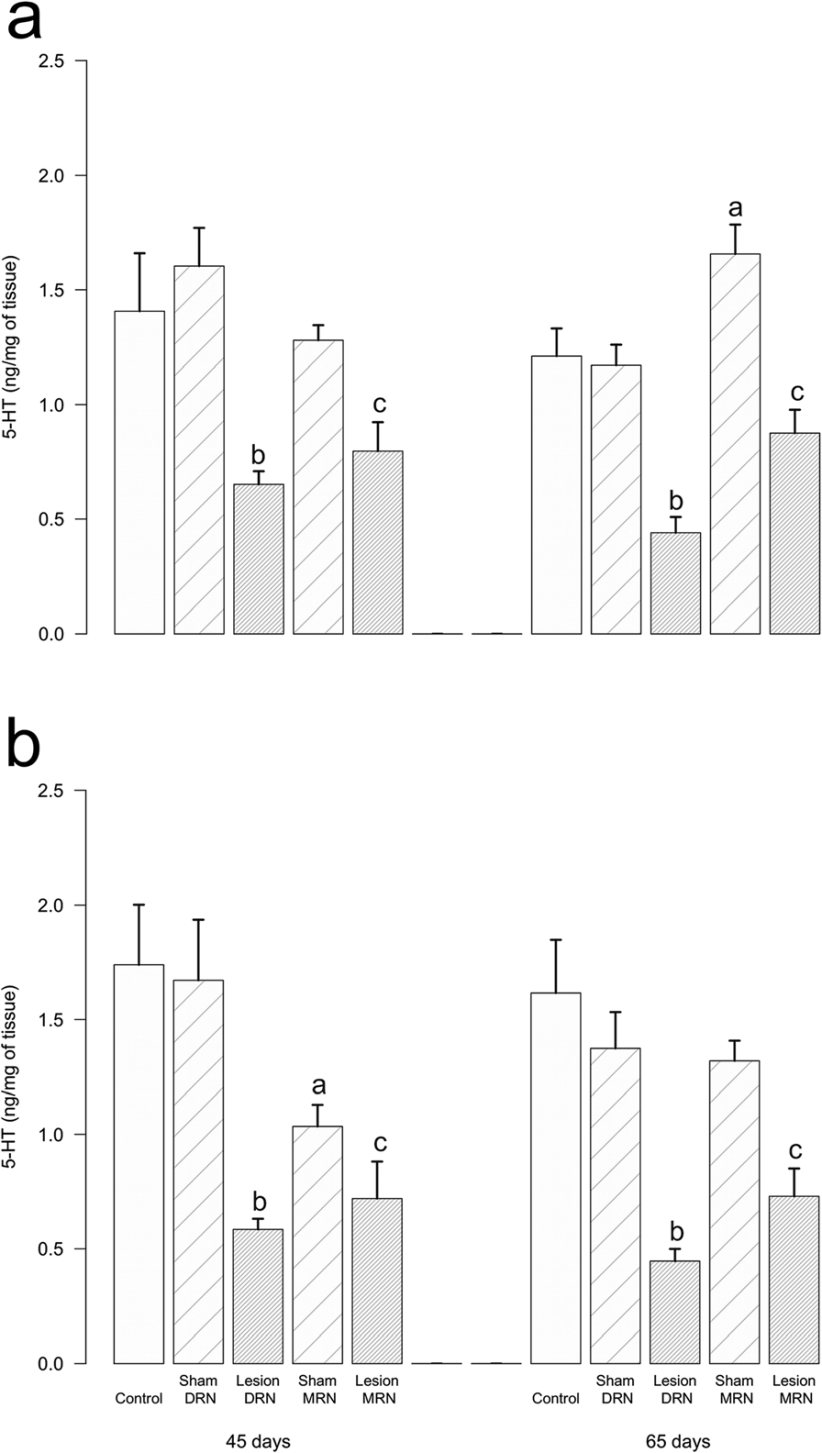
Production of serotonin (5-HT) in the hypothalamus after the lesion of raphe nuclei. Control rats, with sham operation (Sham) or lesion induced by the injection of 5,6-Dihydroxytryptamine (5,6DHT) into the dorsal (DRN) or medial (MRN) raphe nucleus and sacrificed at 45 or 65 days of age; **a** anterior hypothalamus; **b** medial hypothalamus. Values are mean ± s.e.m. a, *P* < 0.05 vs. Control group; b, *P* < 0.05 vs. Sham DRN; c, *P* < 0.05 vs. Sham MRN, Tukey test

Compared to the control group, sham operations to the DRN or MRN did not modify the body, testis, prostate, or seminal vesicle weight (Table 1), nor the noradrenaline and dopamine concentrations in the anterior and medial hypothalamus of animals at either 45 or 65 days-of-age (Table 2). Similarly, no significant differences were observed on the 5-HT concentrations in the anterior or medial hypothalamus of animals with sham operation on the DRN (Fig. 2). Sham operation to the MRN resulted in a decrease of serotonin in the medial hypothalamus at 45 days, and an increase in the anterior hypothalamus of animals sacrificed at 65 days of age (Fig. 2).

**Table 1:** Effects of chemical lesion of the DRN or MRN raphe nuclei on body weight and sexual accesory glands (grams) (Mean ± S.E.M.)

Group		n	Body Weight	Testis	Prostate	Seminal Vesicle
45 Days						
	Control	16	161 ± 5.3	0.847 ± 0.06	0.19 ± 0.04	0.17 ± 0.08
	Sham DRN	20	157 ± 3.4	0.829 ± 0.02	0.14 ± 0.01	0.08 ± 0.01
	Lesion DRN	14	153 ± 3.7	0.771 ± 0.06	0.09 ± 0.01	0.06 ± 0.08
	Sham MRN	11	155 ± 4.2	0.739 ± 0.08	0.16 ± 0.05	0.07 ± 0.01
	Lesion MRN	10	159 ± 4.5	0.718 ± 0.09	0.17 ± 0.08	0.07 ± 0.02
65 Days						
	Control	22	256 ± 4.3	1.426 ± 0.02	0.28 ± 0.02	0.35 ± 0.01
	Sham DRN	20	267 ± 3.4	1.481 ± 0.03	0.23 ± 0.02	0.30 ± 0.02
	Lesion DRN	22	250 ± 5.9	1.403 ± 0.04	0.22 ± 0.02	0.25 ± 0.02
	Sham MRN	10	267 ± 10.6	1.443 ± 0.03	0.36 ± 0.05	0.31 ± 0.02
	Lesion MRN	15	277 ± 4.1	1.521 ± 0.03	0.37 ± 0.19	0.35 ± 0.01

Sham; sham operation; DRN, dorsal raphe nucleus; MRN, medial raphe nucleus

**Table 2:** Effects of chemical lesion of the DRN or MRN raphe nuclei on neurotransmitters concentrations (ng/mg tissue) (Mean ± S.E.M.)

			Anterior		Medial	
Age	Group	n	NA	DA	NA	DA
45 Days						
	Control	11	1.48 ± 0.08	0.37 ± 0.14	1.05 ± 0.07	0.35 ± 0.06
	Sham DRN	11	1.44 ± 0.09	0.32 ± 0.01	1.10 ± 0.07	0.34 ± 0.05
	Lesion DRN	12	1.38 ± 0.18	0.33 ± 0.15	1.33 ± 0.14	0.35 ± 0.04
	Sham MRN	6	1.36 ± 0.17	0.28 ± 0.05	1.37 ± 0.13	0.43 ± 0.09
	Lesion MRN	6	1.47 ± 0.11	0.31 ± 0.06	1.16 ± 0.17	0.40 ± 0.09
65 Days						
	Control	10	1.77 ± 0.09	0.14 ± 0.03	1.63 ± 0.09	0.17 ± 0.02
	Sham DRN	16	1.62 ± 0.09	0.11 ± 0.03	1.71 ± 0.08	0.14 ± 0.02
	Lesion DRN	14	1.71 ± 0.13	0.12 ± 0.01	1.65 ± 0.14	0.18 ± 0.01
	Sham MRN	6	1.90 ± 0.08	0.09 ± 0.07	1.83 ± 0.12	0.21 ± 0.03
	Lesion MRN	11	1.67 ± 0.14	0.08 ± 0.08	1.63 ± 0.13	0.16 ± 0.02

Sham; sham operation; DRN, dorsal raphe nucleus; MRN, medial raphe nucleus; noradrenaline, NA; dopamine, DA

Sham operations to the DRN or MRN did not modify the gonadotropin, progesterone, or testosterone concentrations (Table 3) or sperm concentration, viability, and motility (Table 4).

**Table 3:** Effects of chemical lesion of the DRN or MRN raphe nuclei on hormonal concentrations (Mean ± S.E.M.)

Age at sacrifice	Group	n	FSH (ng/ml of serum)	LH (ng/ml of serum)	Progesterone (ng/ml of serum)	Testosterone (ng/ml of serum)
45 Days						
	Control	16	10.1 ± 0.66	0.57 ± 0.06	16.6 ± 1.9	0.97 ± 0.14
	Sham DRN	20	8.7 ± 0.30	0.37 ± 0.05	12.5 ± 0.91	0.68 ± 0.10
	Lesion DRN	14	5.9 ± 0.87^a,b^	0.33 ± 0.06	10.8 ± 1.2	0.65 ± 0.09
	Sham MRN	11	9.6 ± 0.35	0.68 ± 0.06	12.4 ± 2.1	0.82 ± 0.17
	Lesion MRN	10	8.6 ± 0.56	0.59 ± 0.08	9.2 ± 2.9	0.97 ± 0.15
65 Days						
	Control	22	8.7 ± 0.38	0.58 ± 0.03	8.0 ± 1.1	2.43 ± 0.24
	Sham DRN	20	8.2 ± 0.30	0.60 ± 0.04	9.5 ± 1.3	2.47 ± 0.11
	Lesion DRN	22	5.4 ± 0.35^a,b^	0.42 ± 0.07	7.6 ± 1.5	1.71 ± 0.11
	Sham MRN	10	6.1 ± 0.38	0.75 ± 0.05	4.6 ± 0.8	2.40 ± 0.37
	Lesion MRN	15	6.3 ± 0.28	0.75 ± 0.03	4.2 ± 0.5	1.54 ± 0.31

Mean ± s.e.m; Sham; sham operation; DRN, dorsal raphe nucleus; MRN, medial raphe nucleus

^a^ *p* < 0.05 vs. Control group (two-way ANOVA followed by Tukey's test)

^b^ *p* < 0.05 vs. Sham DRN (two-way ANOVA followed by Tukey's test)

**Table 4:** Effects of chemical lesion of the DRN or MRN raphe nuclei on sperm quality, of rats sacrificed at 65 days-of-age (Mean ± S.E.M.)

Group	n	Sperm concentration (1x10^6^ cells/ml)	Sperm motility (%)	Sperm viability (%)
Control	22	71.1 ± 4.2	58.7 ± 2.9	60.9 ± 1.4
Sham DRN	20	70.2 ± 4.8	63.1 ± 4.7	58.2 ± 2.7
Lesion DRN	22	41.2 ± 2.8*	39.6 ± 4.4*	52.1 ± 3.5
Sham MRN	10	67.0 ± 2.8	55.0 ± 2.3	57.2 ± 1.8
Lesion MRN	15	71.3 ± 2.7	50.4 ± 2.0	61.7 ± 2.0

Mean ± s.e.m; Sham; sham operation; DRN, dorsal raphe nucleus; MRN, medial raphe nucleus

*p < 0.05 vs. Sham DRN (Two way ANOVA followed by Tukey 's test)

**Fig. 3:**
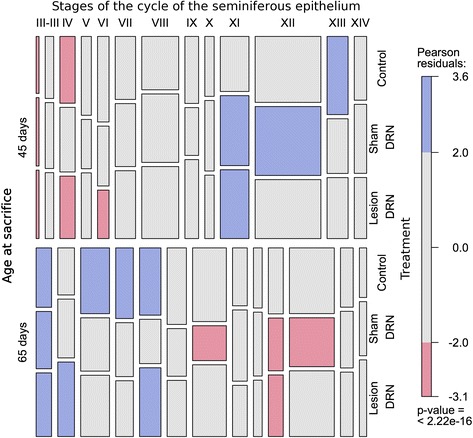
The lesion in the DRN induced changes in the proportions of stages of the cycle of seminiferous epithelium at 45 or 65 days-of age. Mosaic plot for stages of the cycle of seminiferous epithelium, treatment, and age at sacrifice. In the mosaic plot each cell of a contingency table is represented by a rectangle whose area shows the cell frequency or deviation from independence (Friendly, 1994). The area of each cell is proportional to cell frequency. Cells with positive, negative, or no deviation from independence are filled with blue, red, or gray color, respectively (Please see the look up table at right side of the mosaic)

**Fig. 4:**
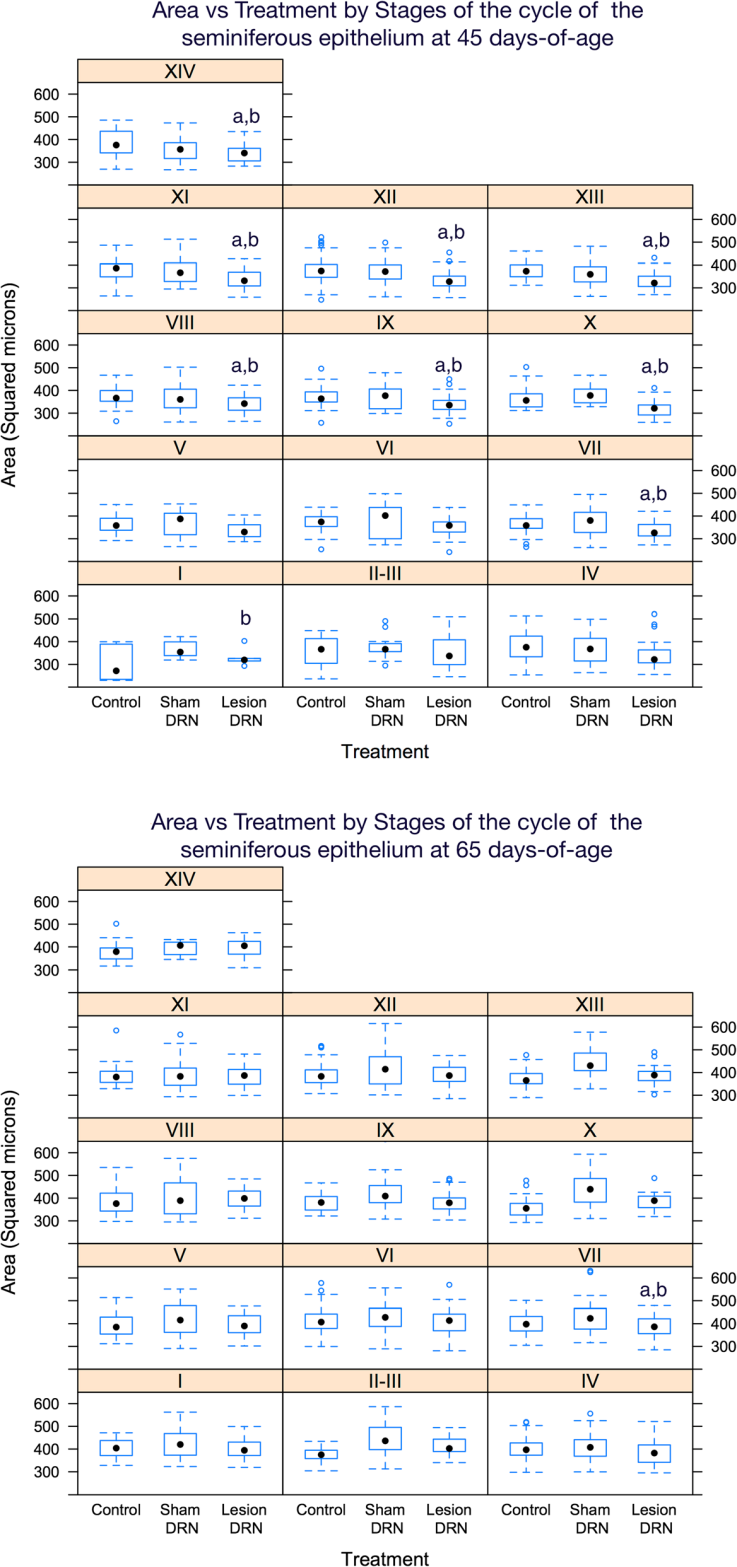
The area of seminiferous tubules is modified by lesion of the DRN at specific stages of the cycle of the seminiferous epithelium. Values are medians (horizontal bar in boxes) with 25 %–75 % interquartile ranges (boxes); dotted vertical lines indicate minimum and maximum values; circles indicate outliers. ^a^P < 0.05 vs Control; ^b^P < 0.05 vs Sham DRN

The sham operation to the DRN, in animals sacrificed at either 45 or 65 days-of-age, did not induce changes in the proportion of stages of the cycle of seminiferous epithelium (Support information 1), but changes in proportion of stages I, XI, and XII were observed between ages (Fig. 3). The area of the seminiferous epithelium (Fig. 4) and the presence of detached cells in the lumen of seminiferous tubules (Table 5), was not changed 15 or 35 days after the sham operation.

**Table 5:** The presence of detached germ cells into the lumen of seminiferous tubules is stage-dependent. Mean number of tubules with germ cells into the lumen 15 or 35 days after the lesion of the DRN (Mean ± S.E.M.)

		Stages of the cycle of the seminiferous epitheliun			
Age at sacrifice	Group	VII	VIII	X	XII
45 Days					
	Control	0	1.0 ± 0.3	0.2 ± 0.1	1.6 ± 0.1
	Sham DRN	1.4 ± 0.5	2.0 ± 0.6	0.4 ± 0.2	2.2 ± 0.5
	Lesion DRN	5.8 ± 1.0^b^	11.0 ± 0.7^a,b^	0.8 ± 0.4	3.8 ± 0.5
65 Days					
	Control	0.2 ± 0.0	1.2 ± 0.5	0	0.8 ± 0.0
	Sham DRN	0.8 ± 0.5	2.0 ± 0.1	0.3 ± 0.1	1.2 ± 0.4
	Lesion DRN	7.0 ± 0.9^a,b^	11.2 ± 1.6^a,b^	1.4 ± 0.2^a,b^	7.6 ± 1.1^a,b^

Sham; sham operation; DRN, dorsal raphe nucleus

^a^P < 0.05 vs. Control; ^b^P < 0.05 vs. Sham DRN (Two way ANOVA followed by Tukey 's test)

Since the sham operation in the MRN modified some the parameters analyzed in this study, the effects of lesion of the DRN or MRN were compared to their respective sham-operated group.

### Effects of the chemical lesion to the DRN or MRN

No significant differences were observed in the weights of body, testis, prostate, or seminal vesicle between respective sham-operated animals and DRN or MRN lesioned-animals sacrificed at either 45 or 65 days of age (Table 1).

The chemical lesion of the DRN or MRN did not modify the noradrenaline or dopamine concentrations in the anterior and medial hypothalamus at 45 or 65 days of age (Table 2). When compared to respective sham operation, lesion of either the DRN or MRN resulted in a significant decrease in serotonin concentration in the anterior and medial hypothalamus of animals sacrificed at 45 or 65 days of age (Fig. 2).

The lesion of the DRN induced a decrease in FSH concentration in rats at 45 or 65 days of age, but the LH, progesterone, and testosterone serum concentrations were unchanged in comparison to respective sham-operated animals. No differences were observed in the FSH, LH, progesterone, and testosterone serum concentrations in the animals with a lesion of the MRN and sacrificed at 45 or 65 days of age (Table 3).

The chemical lesion of the DRN induced a lower sperm concentration and sperm motility, in comparison with those of the respective sham-operated group; but the chemical lesion of the MRN had no effect on these parameters (Table 4). Data of sperm concentration, motility, and viability are not presented as there were no sperm in deferens ducts at 45 days-of-age.

At the testicular level, in the animals with a chemical lesion of DRN there were no changes in proportion of stages of cycle of seminiferous epithelium at either 45 (P = 0.70757) or 65 days-of-age (P = 0.44587) (Additional file 1); however, when age at sacrifice was considered, deviations of independence (P = 2.22 x10^−16^) were observed at stages I, IV, VI, and XI at 45 days-of-age and I, II-III, VI, and XI at 65 days-of age (Fig. 3).

In the animals of 45 days-of-age, the area of seminiferous tubules was lower after chemical lesion of the DRN, as were stages of the cycle of seminiferous epithelium VII-XIV; however, at 65 days-of-age changes were observed only at stage VII (Fig. 4).

Each of the seminiferous tubules evaluated was searched for gross abnormalities. The abnormal presence of detached germ cells, detected in the lumen of seminiferous tubules, was significantly higher, at stages of the cycle of seminiferous epithelium VII, VIII, X and XII in animals with chemical lesion of DRN at 45 and 65 days-of-age (Table 5).

## Discussion

Here we show that the serotonergic system arising from the DRN stimulates FSH secretion and testicular function, whereas the serotonergic system arising from the MRN apparently does not participate in regulation of the secretion of gonadotropins. The reproductive effects, observed 15 days after lesion of the DRN (i.e. at 45 days-of-age,), are partially alleviated at 65 days-of-age.

Our model of chemical lesion of DRN or MRN nuclei by 5,6-DHT effectively damaged serotonergic neurons. This is demonstrated by the fact that serotonin concentrations were low in the anterior and medial hypothalamus, independently of age of sacrifice, whereas noradrenaline and dopamine concentrations were unaffected. Our results are in line with previous reports [29, 40, 41], which reported that the 5,6-DHT is easily incorporated into serotonergic neurons and has the property of autoxidation; this autoxidation generates quinones and free radicals that damage deoxyribonucleic acids, proteins, lipids, and induce the destruction of terminals and axons [40, 41].

The hypothalamus receives serotonergic innervation from the DRN and MRN [10–12]. As observed in the present study, serotonin was detected in the hypothalamus of animals with chemical lesion of the DRN or MRN, indicating that the production of serotonin was not completely eliminated in either group. These results are similar to those reported when a higher dose of the same drug was injected intraventricularly into prepuberal male rats [42]. Two possibilities could explain the presence of serotonin in the hypothalamus of the lesioned animals. First, because we only introduced lesions in DRN or MRN, the presence of the remaining nucleus could be a source of serotonin in the hypothalamus. Second, neurons located in the hypothalamus could produce serotonin, as previously published [reviewed in 20].

The decrease of 5-HT concentration in the hypothalamus of animals with a lesion of the DRN or MRN was maintained from 45 until 65 days-of-age. This results are in line with two facts: 1) 5,6-DHT induce degeneration of serotonergic fibers [43], and 2) degeneration of serotonergic fibers persisted from two weeks to one month [29, 42, 43].

The mechanism by which the MRN sham operation modified the serotonin concentration in the hypothalamus is unknown. This effect could be related with the sectioning of serotonergic fibers, during the introduction of the injection needle. Serotonergic projections of MRN to the ventromedial hypothalamus and preoptic area contain fibers, arising from the left and right side of the nucleus [12]. In all animals with MRN sham operation the needle of microinjection was introduced by the right side. Then, it is possible that the changes in serotonin concentration are related to the surgical trauma of right fibers induced by introduction of the injection needle, and the hyperactivity of undamaged left projections arising from the nucleus.

In the present study, the interruption of serotonergic communication between the DRN and the anterior and medial hypothalamus diminished the concentrations of FSH at 45 and 65 days-of-age. The lack of serotonergic innervation arising from the MRN, did not have an effect on gonadotropin secretion. On this basis, we suggest the existence of two serotonergic systems: a first serotonergic system, arising from the DRN, which innervates the preoptic area, stimulates FSH secretion and sperm production, and does not affect LH secretion; whereas, the second serotonergic system, arising from the MRN, and the main source of serotonin in the mediobasal hypothalamus, does not participate in gonadotropin secretion. This differential effect could-be related to two gonadotropin releasing factors, one for FSH and another for LH, whose existence was previously suggested [44, 45].

Our idea, that the serotonergic system arising from the DRN participates in the modulation of secretion of FSH, is supported by results of a previous report [46], where the treatment of prepuberal male rats with 5-hydroxytryptophan (5-HTP) leads to an increase in serotonin concentration in the hypothalamus and FSH in serum. In the preoptic area, there is communication between serotonergic terminals and GnRH neurons [13, 47, 48]. Also, serotonin in the preoptic area stimulates FSH secretion, which is mediated by the 5-HT2 receptor [49]. The GnRH neurons of mouse have several different serotonin receptors: 5-HT1A, 5-HT3A, and 5-HT4 [50]. Collectively, these studies and our results support the idea that the decrease of FSH induced by the lesion of the DRN leads to the modification of the neuroendocrine mechanism that regulates FSH secretion.

An alternative explanation for the low concentrations of FSH observed in the animals with DNR lesion is that the lack of DRN serotonergic innervation results in the activation of other neural pathways participating in the control of gonadotropin secretion. We suggest that this neural pathway is the serotonergic innervation arising from the MRN; this suggestion is supported by the fact that serotonergic innervation arising from the MRN inhibits the secretion of gonadotropins in the female rat [3, 5]. The DRN and MRN nuclei are anatomically connected [22]; thus, it is possible that the DRN controls the functions of the MRN, as previously suggested [23].

The decrease in sperm count and FSH concentrations observed in our work after chemical lesion of the DRN is in line with reports that support the role of FSH as a factor of germ cell proliferation [51–54]. In the case of prepuberal-pubertal male rat, FSH increases between 30 and 40 days-of-age [55]. FSH is involved in the initiation and maintenance of spermatogenesis; in prepuberal rats, FSH acts in Sertoli cells, and stimulates the secretion of growth factors essential for proliferation and differentiation of germ cells [51, 53, 54]. The decrease of FSH concentrations, induced by hypophysectomy in prepuberal male rats, induces the degeneration of pachytene spermatocytes, secondary spermatocytes, and round spermatids; whereas the treatment of these animals with FSH or FSH plus LH decreased the degeneration of germ cells [52, 56]. The studies mentioned and our results support the idea that the diminution of FSH concentration, induced by the chemical lesion of DRN, has a role in decreasing sperm production.

Spermatogenesis is a multi-factorial regulated process that involves the participation of gonadotropins and androgens, among other factors [51–54, 56]. The results presented herein show that the decreases in the spermatogenesis induced by the lesion of the DRN did not correlate with changes in testosterone levels. This result indicates that factors other than testosterone participate in the modulation of spermatogenesis. One such factor is FSH, which has been previously indicated [54]. Another possibility is that the diminution of FSH concentrations in the animals with a lesion on the DRN could affect the Sertoli cell functions and spermatogenesis. Both testosterone and FSH have a synergistic action on Sertoli cells to maintain spermatogenesis [54]. The action of these hormones is mediated through receptors [57], and FSH facilitates the synthesis of androgen protein binding in Sertoli cells [54, 57]. FSH, together with testosterone, prevents apoptosis and promotes the survival of germ cells, mainly spermatocytes and round spermatids, and are involved in spermiation [51, 53]. Accordingly, this evidence supports the interaction of testosterone and FSH with Sertoli Cells for regulation of spermatogenesis. Based on these lines of evidence, it is possible that the decrease in spermatogenesis in the animals with a lesion in the DRN is related to the diminished action of testosterone, mediated in turn by the diminution on FSH secretion.

The fact that, in the animals with lesion of the MRN, FSH and testosterone concentrations were similar to those observed in the respective sham operated group, indicates that concentrations of these hormones were sufficient to maintain the spermatogenesis.

The fact that, 35 days after the lesion of the MRN, the concentrations of FSH and sperm traits were not altered, indicates that the serotonergic system arising from the MRN, is not involved in the regulation of FSH secretion and spermatogenesis. Possibly, the lack of serotonergic innervation arising in the MRN, is compensated by the presence of serotonergic system arising from the DRN, which exerts a stimulatory role on secretion of FSH and spermatogenesis. This idea is supported by the existence of a neural connection between the DRN and MRN, as previously proposed [22].

The testicular effects of lesion of the DRN are transient. The opposite pattern of proportion of stages of cycle of seminiferous epithelium, with positive or negative deviations of independence, between animals of 45 and 65-days-of-age, and the higher values of the area of seminiferous tubules at 65 days-of-age, indicates the progression of the testicular development. This assumption is supported by the increase in weight of the testis and accessory glands in animals with lesion of the DRN at 65 days-of-age compared with those of 45-days-of-age.

The lesion of the DRN did not affect the concentrations of either LH or sexual steroids. This indicates that functionality of Leydig cells was not altered. Previous evidence supports this assertion that, in the rat, testosterone synthesis occurs mainly in the testis and secretion by the adrenal gland is limited, since the removal of testis resulted in lower testosterone serum levels and adrenalectomy did not modify testosterone serum levels [58].

Together our results show that serotonin arising from the DRN is involved in the modulation of the testis function. Thus the drugs that enhance or inhibit serotonin activity may have implications on reproduction. The use of antidepressants such as selective serotonin reuptake inhibitors (SSRI) induce negative reproductive effects in adult men [59], in woman during pregnancy [60], and male exposed in utero [61]. SSRI and are widely prescribed for children and adolescents, although data regarding their safety are limited [62]. Recently, a detailed description of disruption of the axis hypothalamus-hypophysis-testicle by SSRI was described in mature male zebrafish (Prasad et al., in press [63]), but information on mammal models is still scarce.

## Conclusions

Here we demonstrate that serotonergic system, arising from the DRN and MRN, participates differentially in the modulation of the neuroendocrine mechanisms that regulate testicular function. In light of our results, the use of prescription or abuse drugs which affect the serotonergic system, could impact the activity of DRN and MRN nuclei, and thus affect the male reproductive function.

## Additional file

Additional file 1: The chemical lesion of DRN does not induced changes in proportion of stages of cycle of seminiferous epithelium at either 45 nor 65 days of age. Mosaic plot for stages of the cycle of seminiferous epithelium, treatment, and age at sacrifice. In the mosaic plot each cell of a contingency table is represented by a rectangle whose area shows the cell frequency or deviation from independence (Friendly, 1994). The area of each cell is proportional to cell frequency. Cells with positive, negative, or no deviation from independence are filled with blue, red, or gray color, respectively (Please see the look up table at right side of the mosaic). (TIF 744 kb)
