# Supplementary figures and images for: Dorsal and medial raphe nuclei participate differentially in reproductive functions of the male rat
Source: Reprod Biol Endocrinol. 2015 Dec 8;13:132. doi: 10.1186/s12958-015-0130-0 (PMC4672486; doi:10.1186/s12958-015-0130-0)

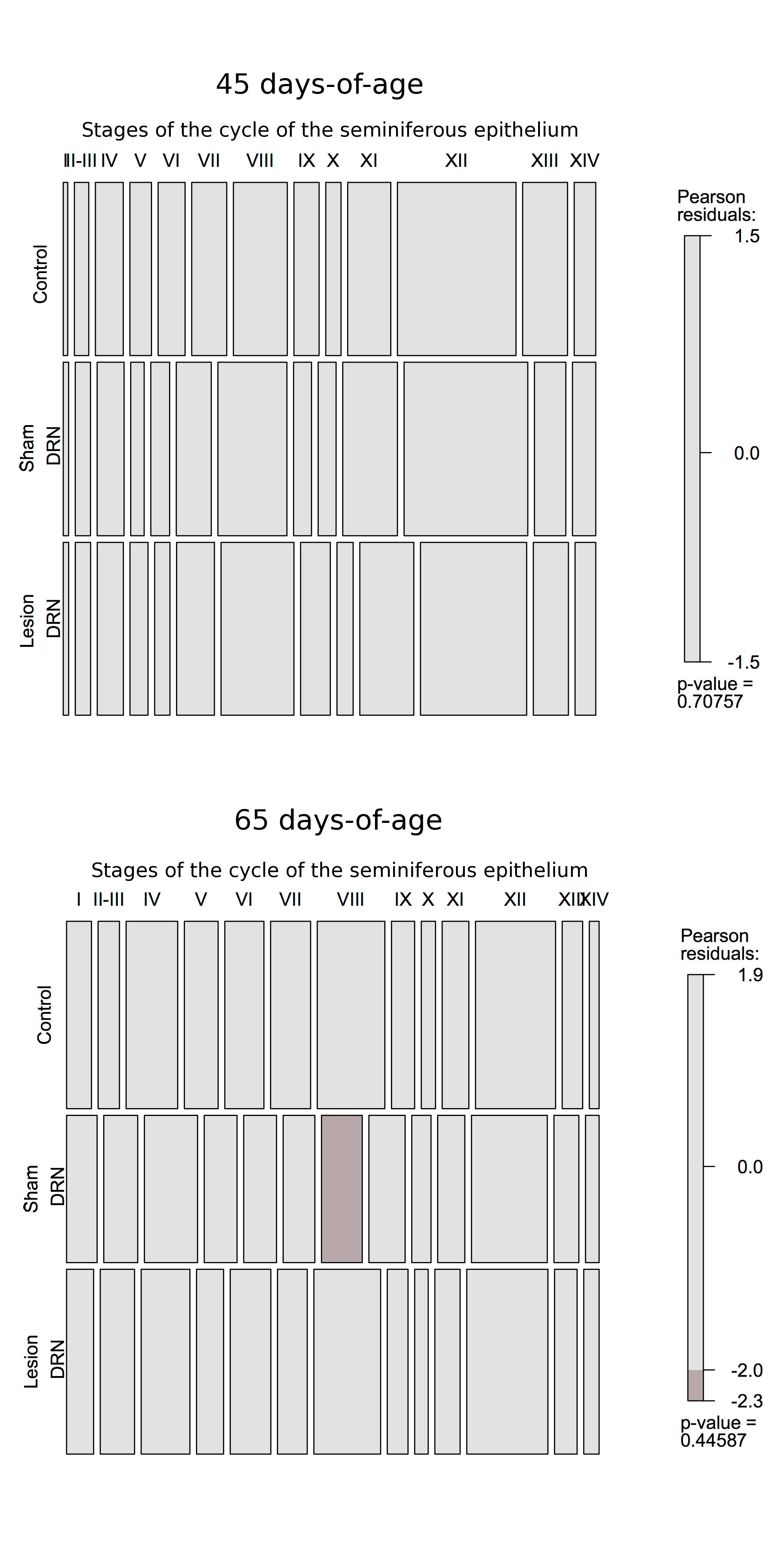

Supplement: Additional file 1: — The chemical lesion of DRN does not induced changes in proportion of stages of cycle of seminiferous epithelium at either 45 nor 65 days of age. Mosaic plot for stages of the cycle of seminiferous epithelium, treatment, and age at sacrifice. In the mosaic plot each cell of a contingency table is represented by a rectangle whose area shows the cell frequency or deviation from independence (Friendly, 1994). The area of each cell is proportional to cell frequency. Cells with positive, negative, or no deviation from independence are filled with blue, red, or gray color, respectively (Please see the look up table at right side of the mosaic). (TIF 744 kb) [file 12958_2015_130_MOESM1_ESM.tif]
